# Secondary Metabolites from *Penicillium pinophilum* SD-272, a Marine Sediment-Derived Fungus

**DOI:** 10.3390/md11062230

**Published:** 2013-06-21

**Authors:** Ming-Hui Wang, Xiao-Ming Li, Chun-Shun Li, Nai-Yun Ji, Bin-Gui Wang

**Affiliations:** 1 Key Laboratory of Experimental Marine Biology, Institute of Oceanology, Chinese Academy of Sciences, Nanhai Road 7, Qingdao 266071, China; E-Mails: wangmh29@yahoo.cn (M.-H.W.); lixmqd@yahoo.com.cn (X.-M.L.); lichunshun@ms.qdio.ac.cn (C.-S.L.); 2 University of Chinese Academy of Sciences, Yuquan Road 19A, Beijing 100049, China; 3 Key Laboratory of Coastal Biology and Bioresource Utilization, Yantai Institute of Coastal Zone Research, Chinese Academy of Sciences, Chunhui Road 17, Yantai 264003, China

**Keywords:** sediment, *Penicillium pinophilum*, secondary metabolite, brine shrimp lethality

## Abstract

Two new secondary metabolites, namely, pinodiketopiperazine A (**1**) and 6,7-dihydroxy-3-methoxy-3-methylphthalide (**2**), along with alternariol 2,4-dimethyl ether (**3**) and l-5-oxoproline methyl ester (**4**), which were isolated from a natural source for the first time but have been previously synthesized, were characterized from the marine sediment-derived fungus *Penicillium pinophilum* SD-272. In addition, six known metabolites (**5**–**10**) were also identified. Their structures were elucidated by analysis of the NMR and mass spectroscopic data. The absolute configuration of compound **1** was determined by experimental and calculated ECD spectra. Compound **2** displayed potent brine shrimp (*Artemia salina*) lethality with LD_50_ 11.2 μM.

## 1. Introduction

**Figure 1:**
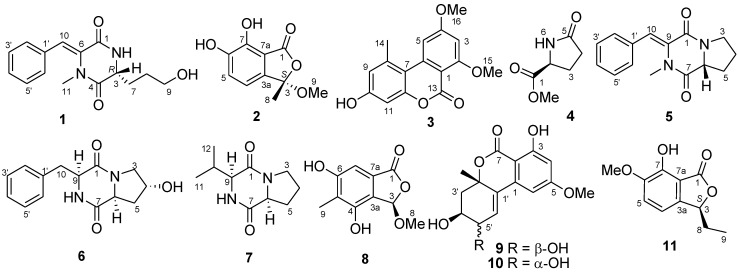
Structures of the isolated compounds **1**–**10** and the reference compound **11**.

Marine fungi are known to be a prolific source of biologically active natural products which might be useful for drug discovery [1,2]. As a special ecosystem, marine sediment provides an abundant of fungal resources, which yielded various secondary metabolites with novel structures and interesting biological activities [3,4,5]. In the course of our search for bioactive metabolites from marine-derived fungi [6,7,8,9,10,11], a strain of *Penicillium pinophilum* SD-272, which was isolated from sediment samples collected from the estuary of the Pearl River in South China Sea, attracted our attention. The EtOAc extract of the culture extract yielded a new diketopiperazine derivative, pinodiketopiperazine A (**1**), a new phthalide derivative, 6,7-dihydroxy-3-methoxy-3-methylphthalide (**2**), as well as alternariol 2,4-dimethyl ether (**3**) [12,13] and l-5-oxoproline methyl ester (**4**) [14], which were isolated from a natural source for the first time but have been previously synthesized (Figure 1). In addition, six known compounds (**5**–**10**) were also isolated and identified. We present herein the isolation, structural elucidation, absolute configuration determination, and biological activity of these compounds.

## 2. Results and Discussion

### 2.1. Structure Elucidation of the New Compounds

**Figure 2:**
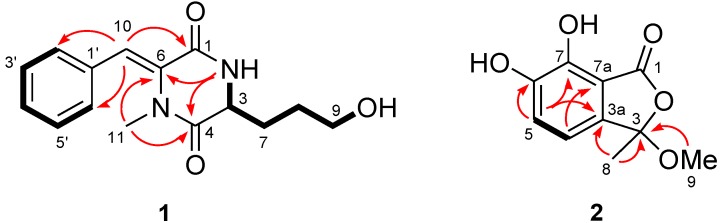
Key COSY (bold lines) and HMBC (arrows) correlations for compounds **1** and **2**.

Compound **1** was obtained as yellowish oil and HRESIMS data established its molecular formula as C_15_H_18_N_2_O_3_, indicating eight degrees of unsaturation. In the ^1^H NMR spectrum (Table 1), signals for a mono-substituted phenyl group (H-2′–H-6′) and a tri-substituted double bond (H-10) were presented, as well as a nitrogenated or oxygenated methine (H-3), three methylenes (H-7–H-9), and a tertiary methyl (H-11) group. The ^13^C and DEPT NMR spectroscopic data (Table 1) exhibited the presence of 15 carbon signals which were further classified into one methyl, three methylenes (with one oxygenated), seven methines, and four quaternary (with two amide ester carbonyl and two olefinic quaternary) carbon atoms. The ^1^H–^1^H COSY spectrum (Figure 2) revealed two spin systems corresponding to two substructures, with the first one being a phenyl group which connected to the double bond as evidenced by the HMBC correlation from H-10 to C-2′/C-6′ (Figure 2). The second substructure being started from an exchangeable NH proton (NH-2), followed by a methine proton H-3, which connected to a straight chain consisting of three methylene groups (CH_2_-7–CH_2_-9) and terminated with an OH group (Figure 2). The connection of the above substructures in **1** as mentioned above was established by detailed inspection of the key HMBC correlations from H-10 to C-1, from H_3_-11 to C-4 and C-6, and from NH-2 to C-4 and C-6. The structure of compound **1** was therefore elucidated as shown in Figure 1. A trivial name pinodiketopiperazine A was assigned to this compound.

**Table 1:** ^1^H- and ^13^C-NMR data of compound **1** in DMSO-*d*_6_^a^.

No.	δ_C_	δ_H_ ( *J* in Hz)	No.	δ_C_	δ_H_ ( *J* in Hz)
1	162.5, C		9	60.1, CH_2_	3.40, m
2-NH		8.62, d (2.8)	10	118.1, CH	6.97, s
3	55.0, CH	3.94, m	11	34.5, CH_3_	2.73, s
4	167.9, C		1′	133.8, C	
6	132.4, C		2′/6′	129.2, CH	7.42, d (7.5)
7	30.6, CH_2_	a 1.79, m	3′/5′	128.3, CH	7.30, t (7.5)
		b 1.71, m	4′	128.1, CH	7.34, t (7.5)
8	28.0, CH_2_	1.51, m	OH		4.48, br s

^a^ Measured at 500 MHz for ^1^H and 125 MHz for ^13^C.

**Figure 3:**
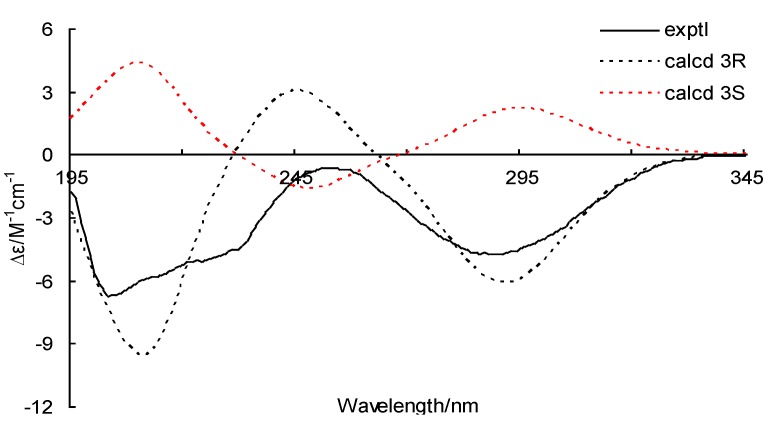
Experimental and calculated electronic circular dichroism (ECD) spectra of **1a** (3*R*) and **1b** (3*S*).

**Figure 4:**
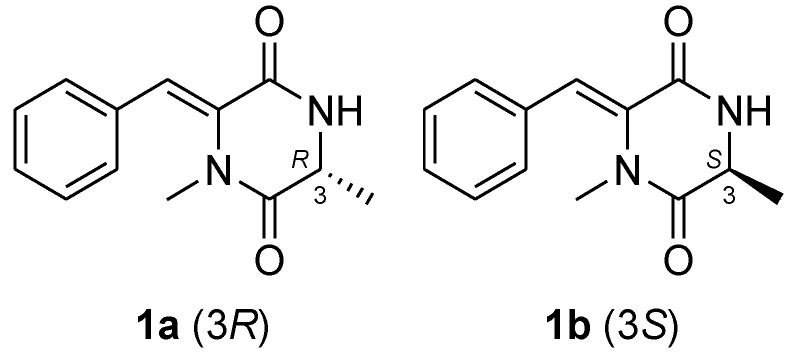
Model compounds **1a** and **1b** for ECD calculations.

To determine the absolute configuration of **1**, the electronic circular dichroism (ECD) was experimentally recorded and it showed negative Cotton effects at 203 and 288 nm (Figure 3). The theoretical ECD was then calculated using the time-dependent DFT (density function theory) method at the B3LYP/6-31G(d) level and was compared with that of the experimental data. The side chain at C-3 in **1** was truncated to a methyl (**1a** and **1b**) (Figure 4) since it contributes little to the ECD spectrum [15,16]. The results showed that, although a slightly peaked shift was observed, the calculated curve of **1a** (with 3*R*-configuration) was in good agreement with that of the experimental one (Figure 3), which indicated that the absolute configuration at C-3 of **1** was *R*.

Compound **2** was obtained as white amorphous powder and its molecular formula C_10_H_10_O_5_ was determined on the basis of the positive HRESIMS. Detailed analysis of the NMR spectroscopic data (Table 2) revealed that the structure of **2** was similar to (*S*)-3-ethyl-7-hydroxy-6-methoxyphthalide (**11**, Figure 1) [17]. However, the C-6 OMe group in **11** was replaced by OH group in **2** as evidenced by the fact that the resonances corresponding to the C-6 OMe group at δ_H_ 3.84/δ_C_ 56.2 in the NMR spectra of **11** disappeared in that of **2**. In addition, the proton signals at δ_H_ 5.51 (H-3), 1.83/2.30 (H-8), and 0.95 (H-9) as well as the carbon signals at δ_C_ 83.3 (CH, C-3), 25.9 (CH_2_, C-8), and 9.1 (CH_3_, C-9) in the NMR spectra of **11** were missing in that of **2**. Instead, signals resonating at δ_C_ 108.6 (C) for a ketal carbon (C-3), at δ_H_ 1.74/δ_C_ 25.6 for a methyl group (CH_3_-8), and at δ_H_ 2.94/δ_C_ 52.4 for an OMe group (OCH_3_-9), were observed in the NMR spectra of **2** (Table 2). The HMBC correlations from H-8 and H-9 to C-3 and from H-8 to C-3a supported the above deduction (Figure 2). The structure of **2** was thus determined as 6,7-dihydroxy-3-methoxy-3-methylphthalide. To unambiguously determine the absolute configuration, compound **2** was also submitted to ECD measurement and calculation. However, no obvious Cotton effect was observed in the experiment. Thus, ECD was not applicable to resolve the absolute configuration of compound **2**. By comparison of its optical rotation ([α]_D_^27^ −8.3, *c* 0.12, CHCl_3_) with that of the (*S*)-3-ethyl-7-hydroxy-6-methoxyphthalide (**11**) ([α]_D_^25^ −71.5, *c* 0.10, CHCl_3_) [17], the *S-*configuration at C-3 was tentatively deduced to this compound.

**Table 2:** ^1^H- and ^13^C-NMR data of compound **2** in DMSO-*d*_6_^a^.

No.	δ_C_	δ_H_ ( *J* in Hz)	No.	δ_C_	δ_H_ ( *J* in Hz)
1	167.6, C		6	146.6, C	
3	108.6, C		7	151.1, C	
3a	132.9, C		7a	114.6, C	
4	121.2, CH	6.84, d (8.5)	8	25.6, CH_3_	1.74, s
5	125.7, CH	7.01, d (8.5)	9	52.4, CH_3_	2.94, s

^a^ Measured at 500 MHz for ^1^H and 125 MHz for ^13^C.

Compounds **3** and **4** were previously reported as synthetic products and were obtained and described here for the first time as naturally occurring fungal metabolites [12,13,14]. The NMR data for compound **3** was not published previously and the fully assigned ^1^H and ^13^C NMR data for this compound were provided in the Experimental Section.

In addition to compounds **1**–**4**, six known secondary metabolites including *N*-methylphenyldehydroalanyl-l-prolin-anhydrid (**5**) [18,19], cyclo-*trans*-4-OH-(d)-Pro-(d)-Phe (**6**) [20], cyclo(d)-Pro-(d)-Val (**7**) [20], rubralide C (**8**) [21], 5′-epialtenuene (**9**) [22] and altenuene (**10**) [22] (Figure 1), were also isolated, and their structures were elucidated by comparing the NMR data with those of literature reports [18,19,20,21,22].

### 2.2. Biological Activities of the Isolated Compounds

The isolated compounds **1**–**10** were evaluated for the brine shrimp (*Artemia salina*) lethal activity and antibacterial activity. Among them, compound **2** was found to possess potent lethality with LD_50_ 11.2 μM, which is more active than that of the positive control colchicine (with LD_50_ 92.1 μM), but the other tested compounds only displayed weak activity. In the antibacterial assay, compounds **1**, **3**, **5**–**8**, and positive control chloromycetin displayed inhibitory activity against *Escherichia coli*, causing the 10.0, 9.0, 8.0, 8.0, 7.0, 10.0, and 15.0 mm zones of inhibition at 20 μg/disk, respectively.

## 3. Experimental Section

### 3.1. General

NMR Spectra was recorded on a Bruker Avance-500 MHz spectrometer (500 MHz for ^1^H and 125 MHz for ^13^C) and chemical shifts were recorded as δ values. Low and high ESI-Mass spectra were performed on a VG Auto spec 3000 spectrometer. Optical rotations were obtained on Optical Activity A-55 polarimeter. UV Spectra were measured in methanol on a Lengguang Gold S54 spectrophotometer. Silica gel (SiO_2_; 100–200 mesh, 200–300 mesh and GF_254_) for column chromatography and preparative thin-layer chromatography were produced by Qingdao Haiyang Chemical Group Corporation. RP-18 reverse-phase silica gel (40–63 µm) and Sephadex LH-20 were purchased from the Merck Corporation. All solvents were distilled to use.

### 3.2. Fungal Material

The fungal strain *Penicillium pinophilum* SD-272 was isolated from the sediment sample collected from the estuary of the Pearl River in South China Sea, in October 2010. The fungal identification was achieved by analysis of the ITS region of its rDNA as described previously [23]. The sequence data obtained from the fungus has been submitted to GeneBank with accession number KC 427134. A voucher specimen was stored at the Key Laboratory of Experimental Marine Biology of the Institute of Oceanology, Chinese Academy of Sciences.

### 3.3. Fermentation

One hundred 1000-mL Erlenmeyer flasks, each contains 300 mL liquid medium (sucrose 2%, peptone 0.5%, yeast extract 0.3%, monosodium glutamate 1%, mannitol 2%, potato flour 0.4%, seawater, pH 6.5), were sterilized at 116 °C for 20 min and cooled to room temperature subsequently. A piece of mycelium (size 3 cm^2^) growing on malt agar plate was inoculated into 1000-mL Erlenmeyer flask. Static fermentation was then conducted at 28 °C for 35 days.

### 3.4. Extraction and Isolation

The culture broth was filtrated using filter paper and separated into mycelia and culture broth. The air-dried mycelia were immersed in acetone-H_2_O (4:1) with ultrasonic processor for 20 min and then extracted three times with ethyl acetate, while the culture broth was stirred for three times with ethyl acetate and then concentrated to obtain an organic extract. Since the two extract show similar HPLC and TLC profiles, they were combined to afford a crude extract (50.2 g) for further purification.

The crude extract was subjected to vacuum liquid chromatography (VLC) on silica gel eluting with step solvents of increasing polarity (from petroleum ether to MeOH) to yield 9 fractions (Fr.1–Fr.9). Fr.1 was subjected to Sephadex LH-20 (acetone) and preparative-TLC to afford **2** (4.5 mg). Fr.3 was separated by column chromatography (CC) on Lobar LiChroprep C_18_ eluting with MeOH–H_2_O gradient to give five sub-fractions (Fr.3.1–Fr.3.5). Fr.3.3 was then chromatographed on silica gel eluting with CHCl_3_–MeOH gradient (60:1) and further purified by Sephadex LH-20 (MeOH) to afford **3** (9.4 mg), **5** (7.1 mg), and **8** (5.1 mg). Fr.4 was also further separated by CC on silica gel to give five subfractions (Fr.4.1–Fr.4.5). Fr.4.2 was purified by CC on Sephadex LH-20 (MeOH) and by semi-preparative HPLC using MeOH–H_2_O gradient (50:50) to yield **1** (4.1 mg, *t_R_* 25.6 min), **7** (7.0 mg, *t_R_* 18.1 min), and **6** (28.7 mg, *t_R_* 20.8 min). Fr.4.4 was subjected to CC on silica gel using CHCl_3_–MeOH gradient (50:1) and purified by Sephadex LH-20 (acetone) to get **9** (10.2 mg), **10** (5.4 mg), and **4** (14.6 mg).

Compound **1**: yellowish oil; [α]_D_^27^ −224 (*c* 0.25, MeOH); UV (MeOH) λ_max_ (log ε) 200 (4.31), 291 (4.09) nm; CD λ_max_ (∆ε) 195 (−4.36), 203 (−16.79), 252 (−1.56), 288 (−11.82) nm; ^1^H and ^13^C NMR data, see Table 1; HRESIMS *m/z* 275.1398 [M + H]^+^ (calcd for C_15_H_19_N_2_O_3_, 275.1390), 297.1216 [M + Na]^+^ (calcd for C_15_H_18_N_2_O_3_Na, 297.1215).

Compound **2**: white amorphous powder; [α]_D_^27^ −8.3 (*c* 0.12, CHCl_3_); UV (MeOH) λ_max_ (log ε) 215 (4.54), 237 (4.05), 331 (4.01) nm; ^1^H and ^13^C NMR data, see Table 2; HRESIMS *m/z* 211.0597 [M + H]^+^ (calcd for C_10_H_11_O_5_, 211.0607).

Compound **3**: white amorphous powder; UV (MeOH) λ_max_ (log ε) 200 (3.69), 217 (3.58), 255 (3.75), 288 (3.27), 300 (3.26), 333 (3.29) nm; ^1^H NMR data (at 500 MHz in DMSO-*d*_6_): δ_H_ 6.69 (s, H-3), 7.24 (s, H-5), 6.65 (s, H-9), 6.53 (s, H-11), 10.25 (s, OH-10), 2.73 (s, H-14), 3.89 (s, H-15), 3.94 (s, H-16); ^13^C NMR data (at 125 MHz in DMSO-*d*_6_): δ_C_ 102.0 (C, C-1), 163.5 (C, C-2), 97.3 (CH, C-3), 164.5 (C, C-4), 102.1 (CH, C-5), 139.8 (C, C-6), 116.6 (C, C-7), 137.7 (C, C-8), 116.6 (CH, C-9), 158.4 (C, C-10), 100.7 (CH, C-11), 153.5 (C, C-12), 156.1 (C, C-13), 24.8 (CH_3_, C-14), 56.1 (CH_3_, C-15), 55.6 (CH_3_, C-16). HRESIMS *m/z* 287.0918 [M + H]^+^ (calcd for C_16_H_15_O_5_, 287.0920).

### 3.5. Computational Details

**Figure 5:**
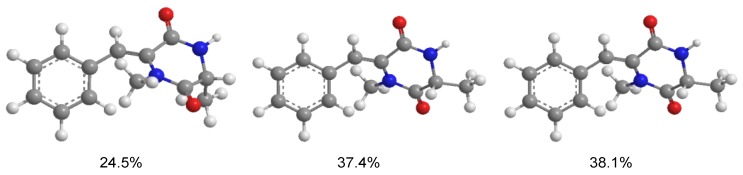
Conformers with populations of **1a** (in MeOH).

**Figure 6:**
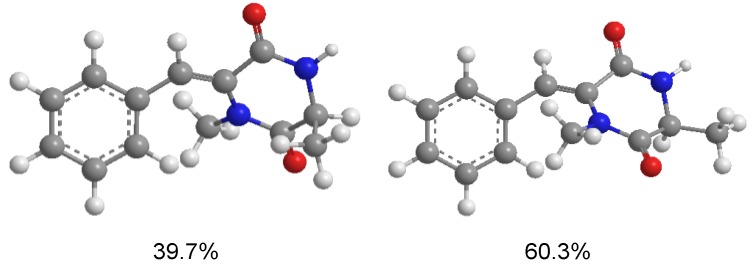
Conformers with populations of **1b** (in MeOH).

Conformational searches for **1a** and **1b** were performed via the Dreiding force field in MarvinSketch regardless of rotations of methyl and hydroxyl groups [24], the geometries of which were further optimized at the B3LYP/6-31G (d) level in methanol to give the conformers (three for **1a** and two for **1b**, see Figure 5 and Figure 6, respectively) within a 3 kcal/mol energy threshold from the global minimum without vibrational imaginary frequencies. The optimized conformers were then subjected to the calculations of ECD spectra using the time-dependent density functional theory (TD-DFT) method at the B3LYP/6-31G(d) level, which were drawn via SpecDic software with sigma = 0.25 and UV shift = −20 nm (magnified by 0.5 times) and weighted by Boltzmann distribution (Figure 3), respectively [25]. All the above calculations were performed with the integral equation formalism variant polarizable continuum model (IEF-PCM) as implemented in Gaussian 09 [26].

### 3.6. Antibacterial Assays

The experiments were performed using the disk diffusion method [27]. Chloromycetin was used as positive control.

### 3.7. Brine Shrimp *(Artemia salina)* Lethality Assay

The assay was carried out following the literature [28]. Colchicine and DMSO were used as positive and negative controls, respectively.

## 4. Conclusions

Two new compounds including pinodiketopiperazine A (**1**) and 6,7-dihydroxy-3-methoxy-3-methylphthalide (**2**), as well as two new naturally occurring compounds including alternariol 2,4-dimethyl ether (**3**) and l-5-oxoproline methyl ester (**4**), together with six known compounds (**5**–**10**) were isolated and identified from the marine sediment-derived fungus *Penicillium pinophilum* SD-272. The absolute configuration of compound **1** was determined by experimental and calculated ECD spectra. Compound **2** was found to possess potent lethality with LD_50_ 11.2 μM, while compounds **1**, **3**, and **5**–**8** displayed inhibitory activity against *Escherichia coli*, causing the 10.0, 9.0, 8.0, 8.0, 7.0, and 10.0 mm zones of inhibition, respectively.

## Supplementary Files

Supplementary File 1 Supplementary Information (PDF, 803 KB)
