# Supplementary material for: Secondary Metabolites from Penicillium pinophilum SD-272, a Marine Sediment-Derived Fungus
Source: Mar Drugs. 2013 Jun 21;11(6):2230–8. doi: 10.3390/md11062230 (PMC3721231; doi:10.3390/md11062230)
Supplement: Supplementary File 1 — Supplementary Information (PDF, 803 KB) [file marinedrugs-11-02230-s001.pdf]

Supplementary Information

Figure S1. ^{1}H NMR (500 MHz, DMSO-d_6) spectrum of compound **1**. 2
Figure S2. ^{13}C NMR (125 MHz, DMSO-d_6) and DEPT spectra of compound **1**. 2
Figure S3. ^{1}H–^{1}H COSY spectrum of the compound **1**. 3
Figure S4. HSQC spectrum of compound **1**. 3
Figure S5. HMBC spectrum of compound **1**. 4
Figure S6. ^{1}H NMR (500 MHz, DMSO-d_6) spectrum of compound **2**. 4
Figure S7. ^{13}C NMR (125 MHz, DMSO-d_6) and DEPT spectra of compound **2**. 5
Figure S8. ^{1}H–^{1}H COSY spectrum of the compound **2**. 5
Figure S9. HSQC spectrum of compound **2**. 6
Figure S10. HMBC spectrum of compound **2**. 6
Figure S11. ^{1}H NMR (500 MHz, DMSO-d_6) spectrum of compound **3**. 7
Figure S12. ^{13}C NMR (125 MHz, DMSO-d_6) and DEPT spectra of compound **3**. 7
Figure S13. ^{1}H–^{1}H COSY spectrum of the compound **3**. 8
Figure S14. HSQC spectrum of compound **3**. 8
Figure S15. HMBC spectrum of compound **3**. 9
^{1}H- and ^{13}C-NMR data of compound **4**. 9
^{1}H- and ^{13}C-NMR data of compound **5**. 9
^{1}H- and ^{13}C-NMR data of compound **6**. 9
^{1}H- and ^{13}C-NMR data of compound **7**. 10
^{1}H- and ^{13}C-NMR data of compound **8**. 10
^{1}H- and ^{13}C-NMR data of compound **9**. 10
^{1}H- and ^{13}C-NMR data of compound **10**. 10

Figure S1. ^{1}H NMR (500 MHz, DMSO-d_6) spectrum of compound **1**.

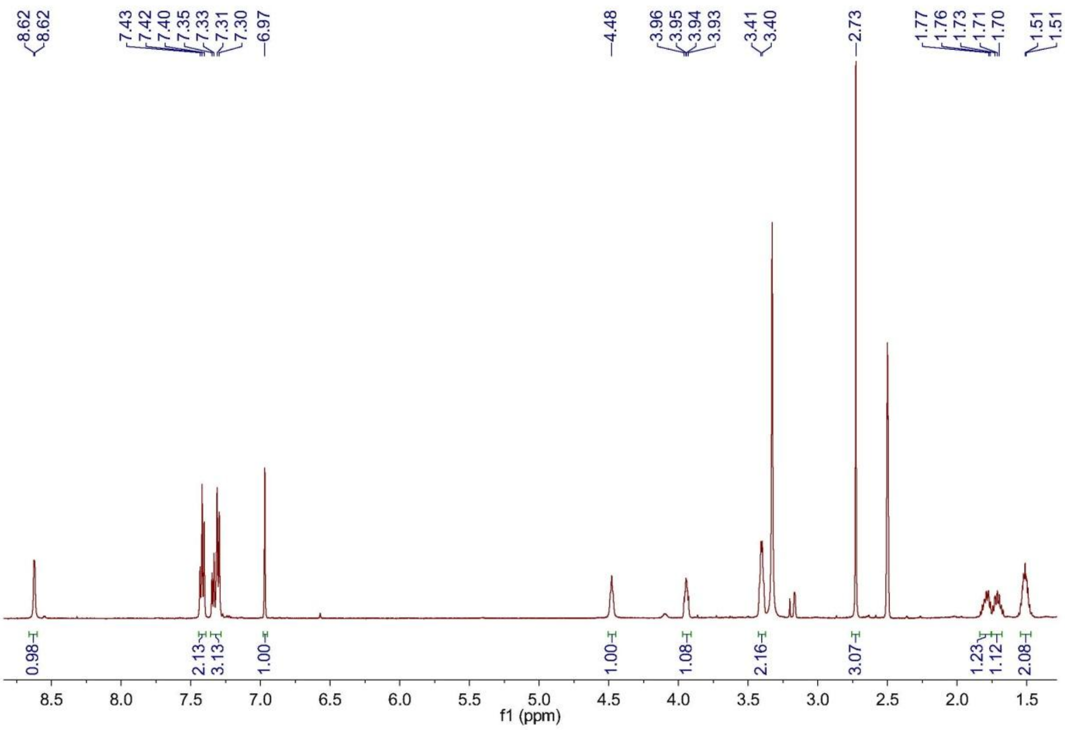

Figure S2. ^{13}C NMR (125 MHz, DMSO-d_6) and DEPT spectra of compound **1**.

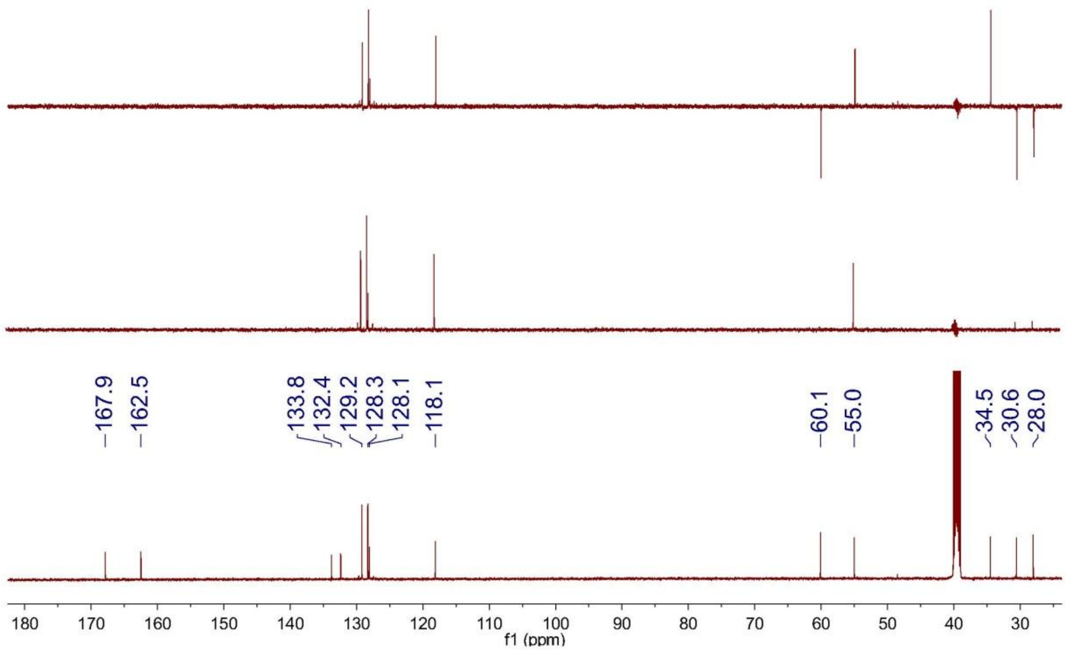

Figure S3. ^{1}H–^{1}H COSY spectrum of the compound **1**.

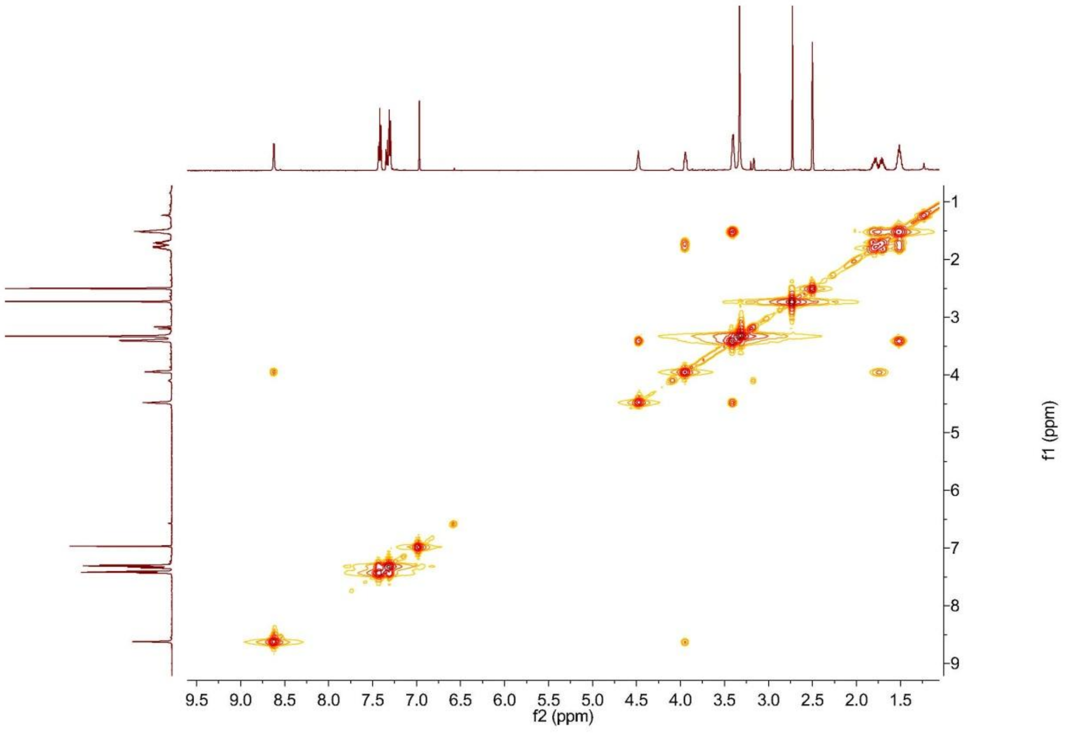

Figure S4. HSQC spectrum of compound **1**.

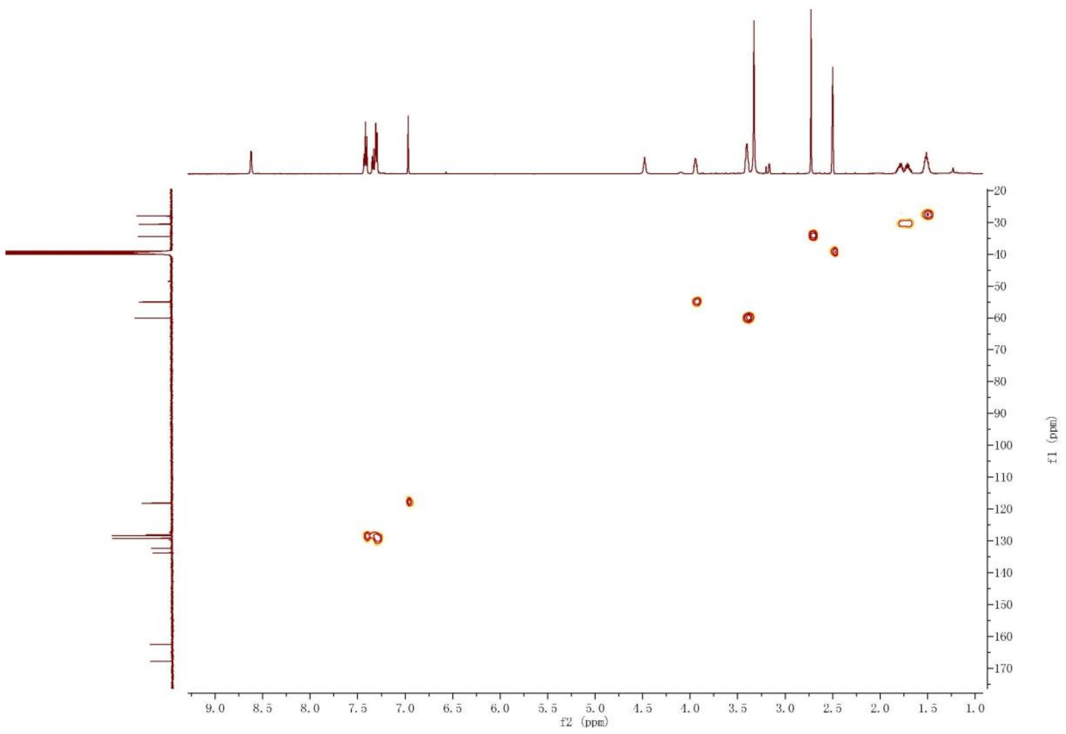

Figure S5. HMBC spectrum of compound **1**.

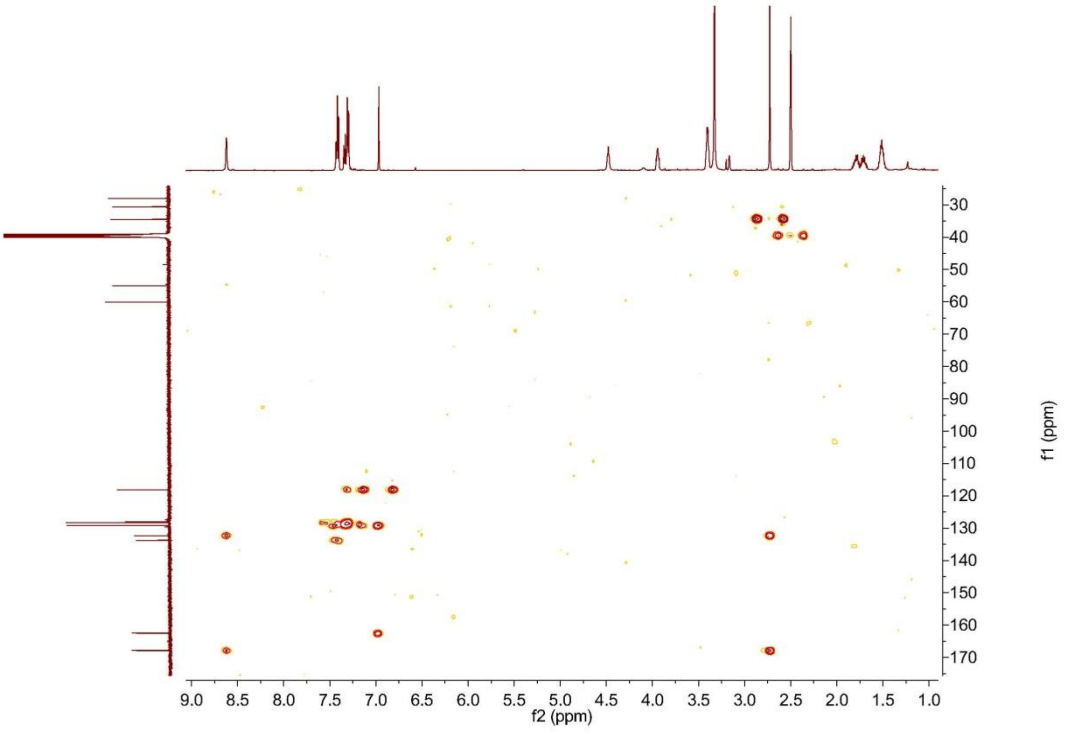

Figure S6. ^{1}H NMR (500 MHz, DMSO-d_6) spectrum of compound **2**.

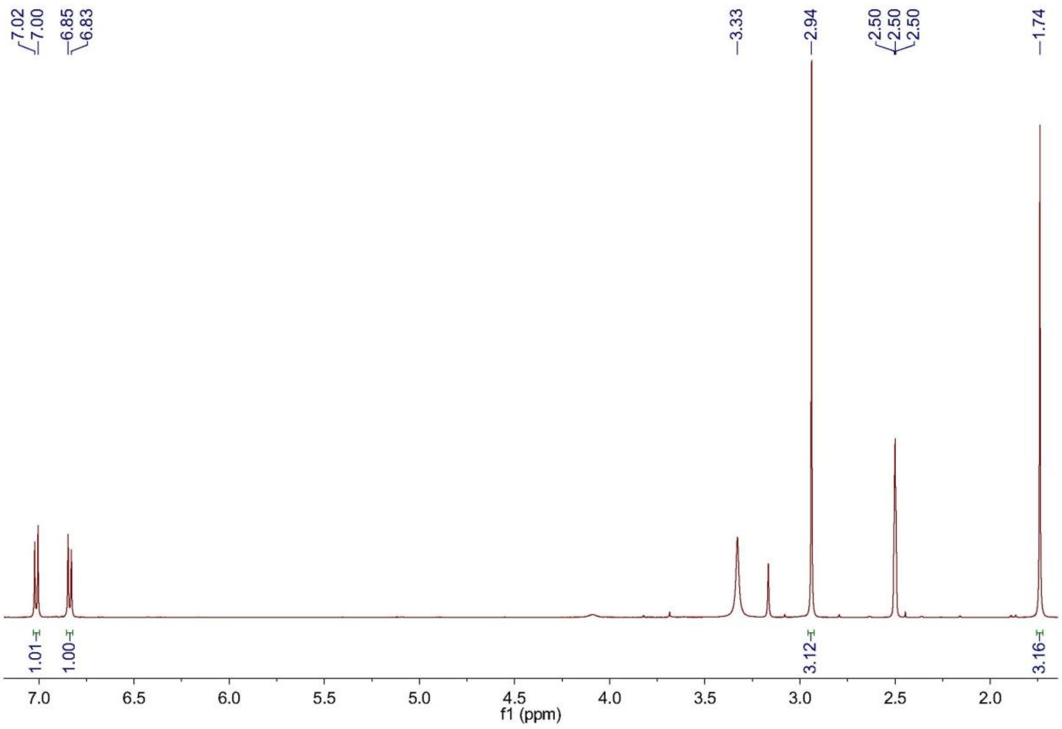

Figure S7. ^{13}C NMR (125 MHz, DMSO-d_6) and DEPT spectra of compound **2.**

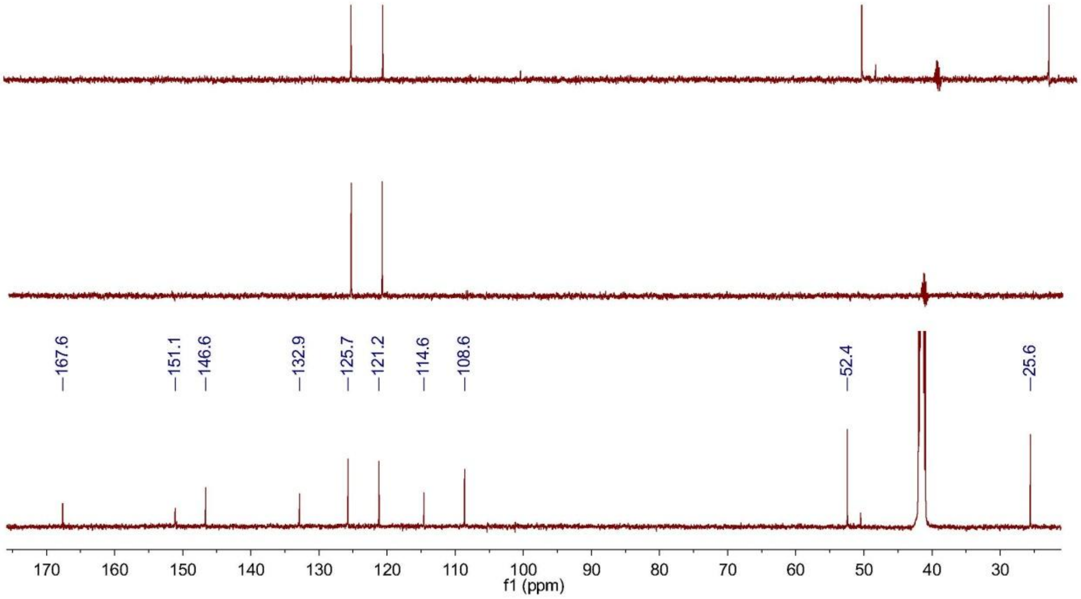

Figure S8. ^{1}H–^{1}H COSY spectrum of the compound **2**.

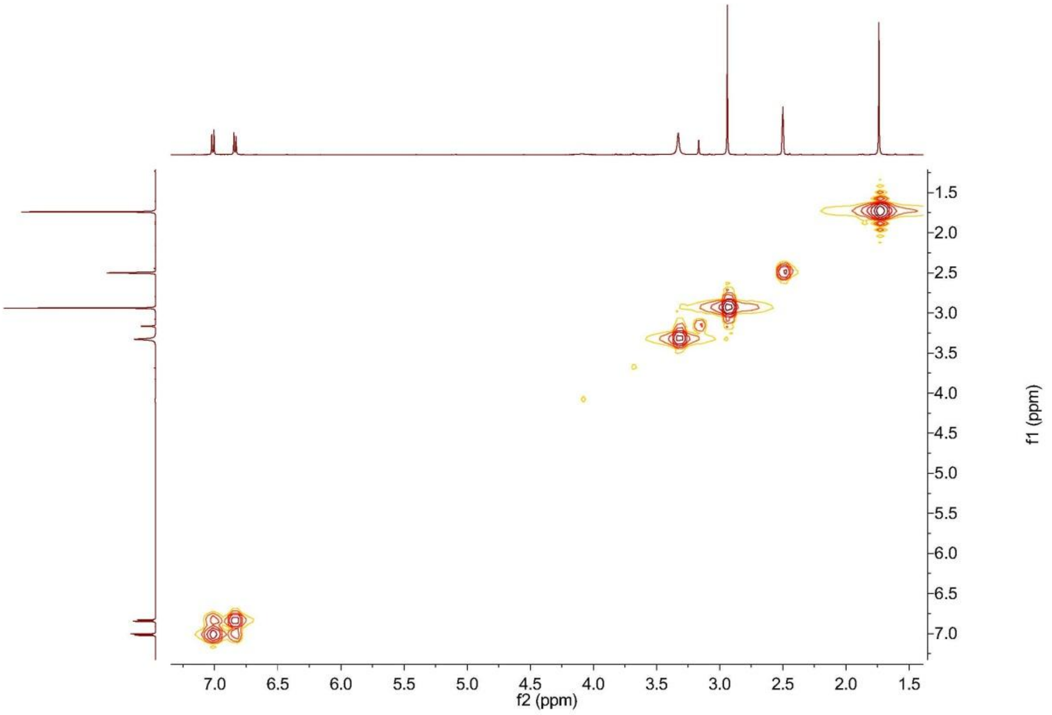

Figure S9. HSQC spectrum of compound **2**.

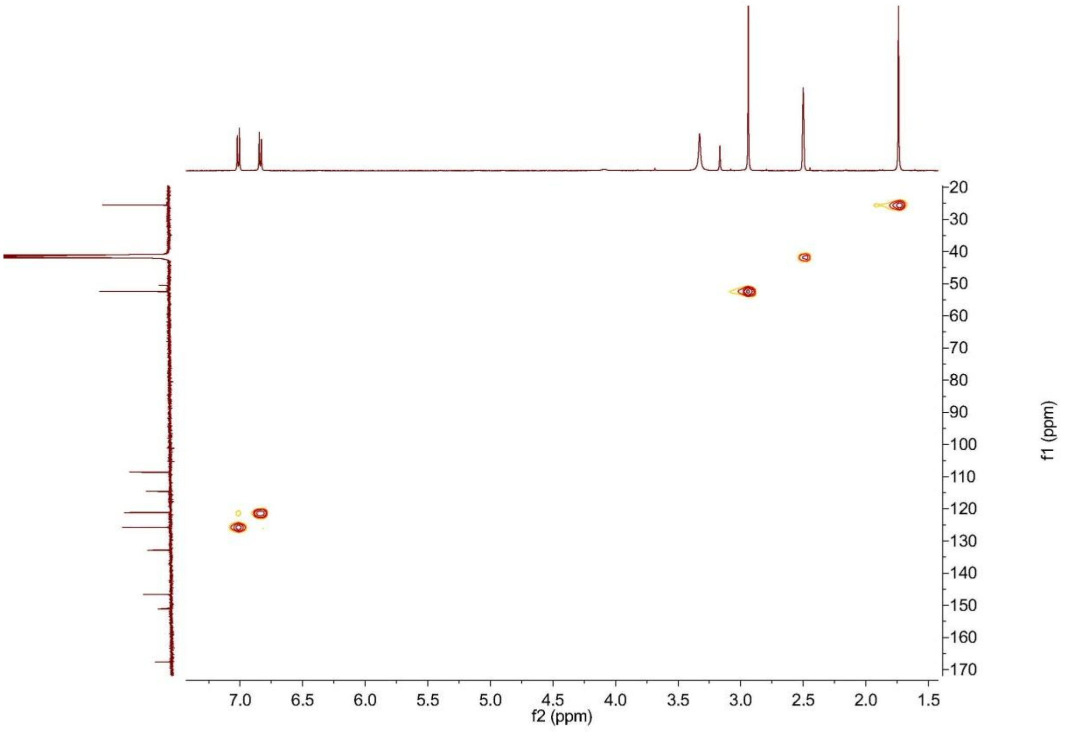

Figure S10. HMBC spectrum of compound **2**.

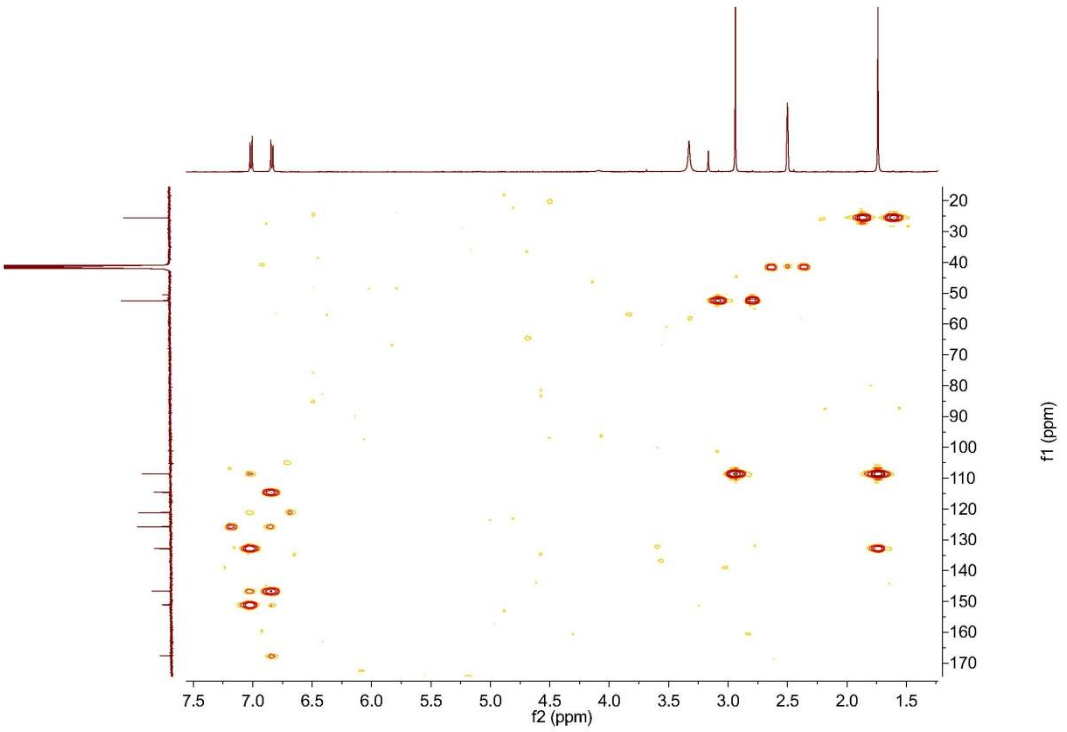

Figure S11. ^{1}H NMR (500 MHz, DMSO-d_6) spectrum of compound **3**.

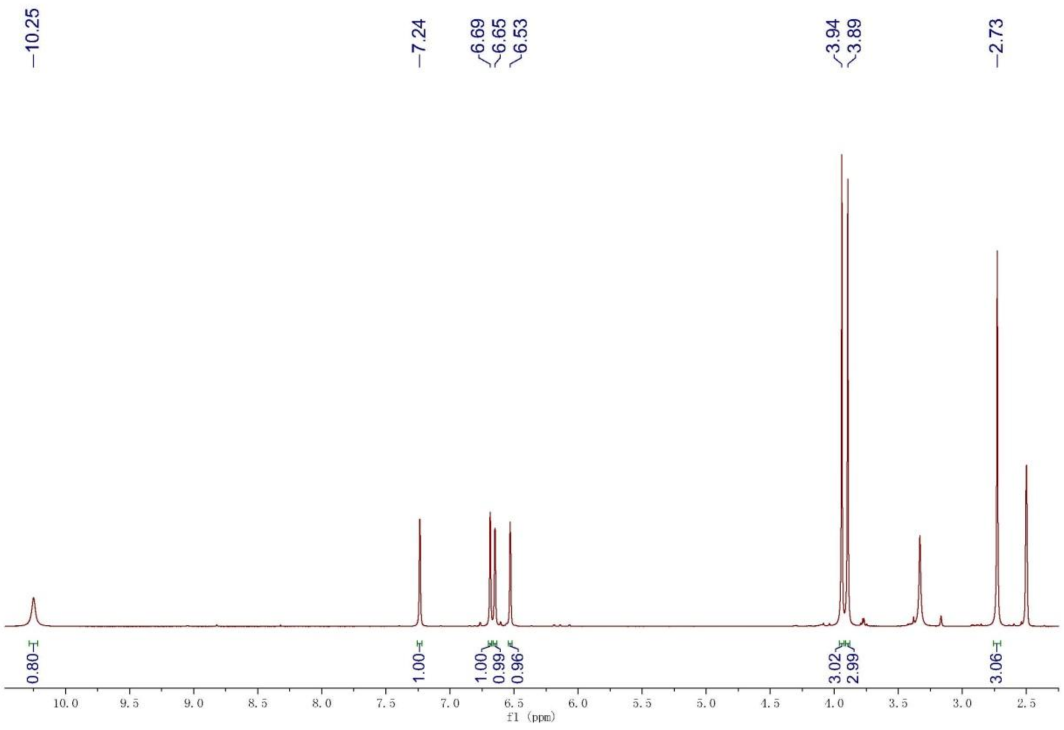

Figure S12. ^{13}C NMR (125 MHz, DMSO-d_6) and DEPT spectra of compound **3**.

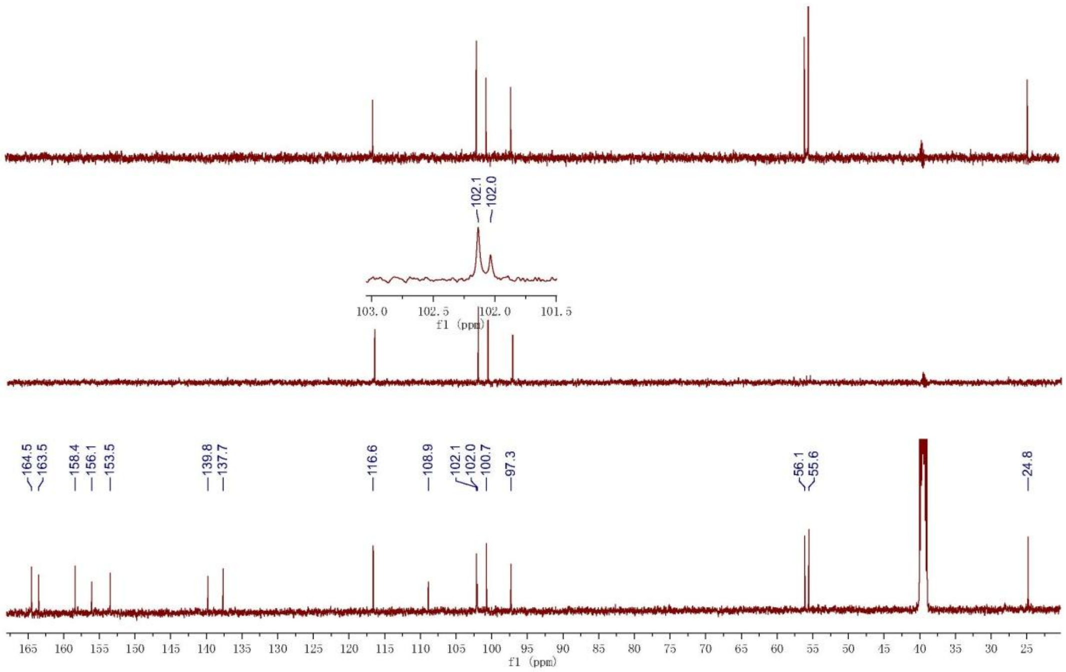

Figure S13. ^{1}H–^{1}H COSY spectrum of the compound **3**.

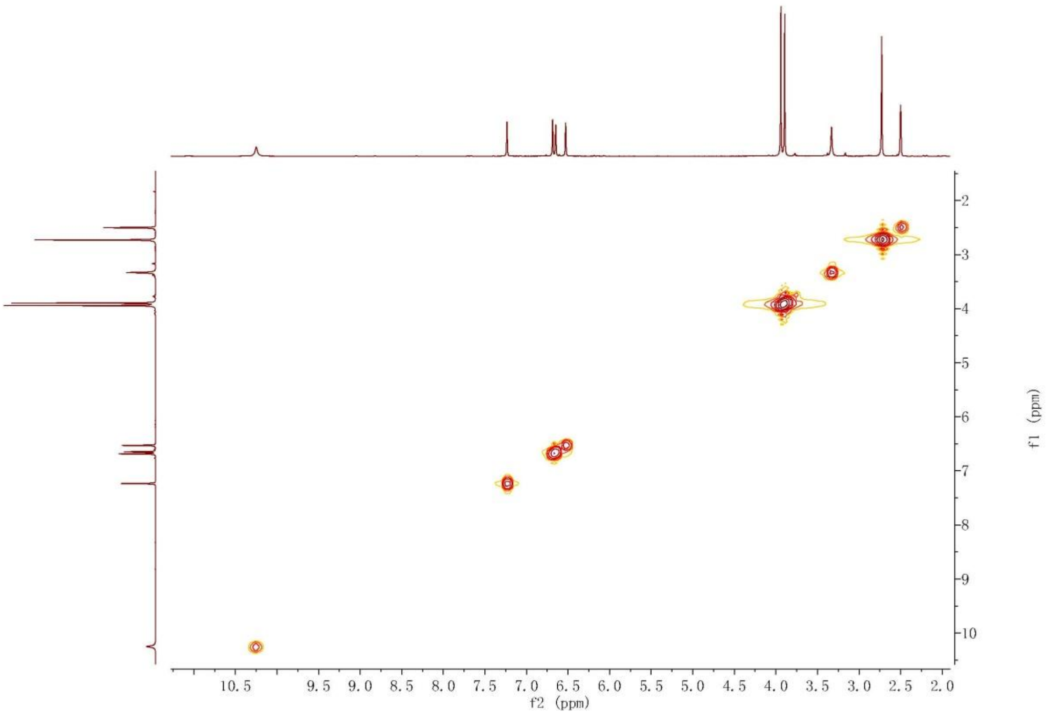

Figure S14. HSQC spectrum of compound **3**.

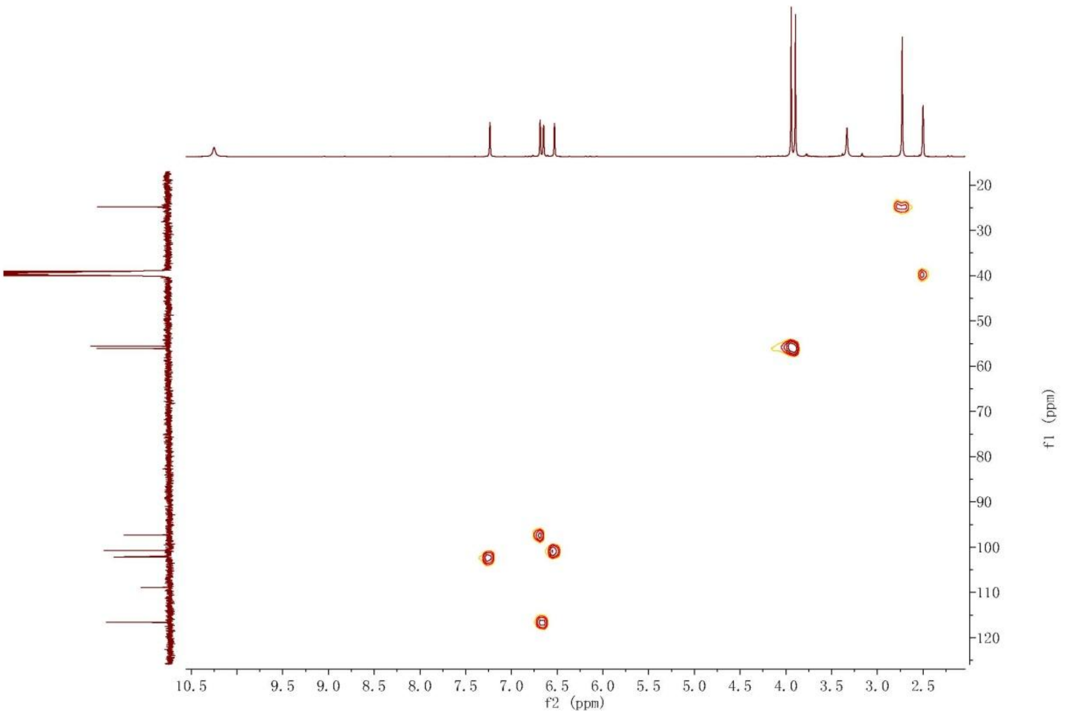

Figure S15. HMBC spectrum of compound **3.**

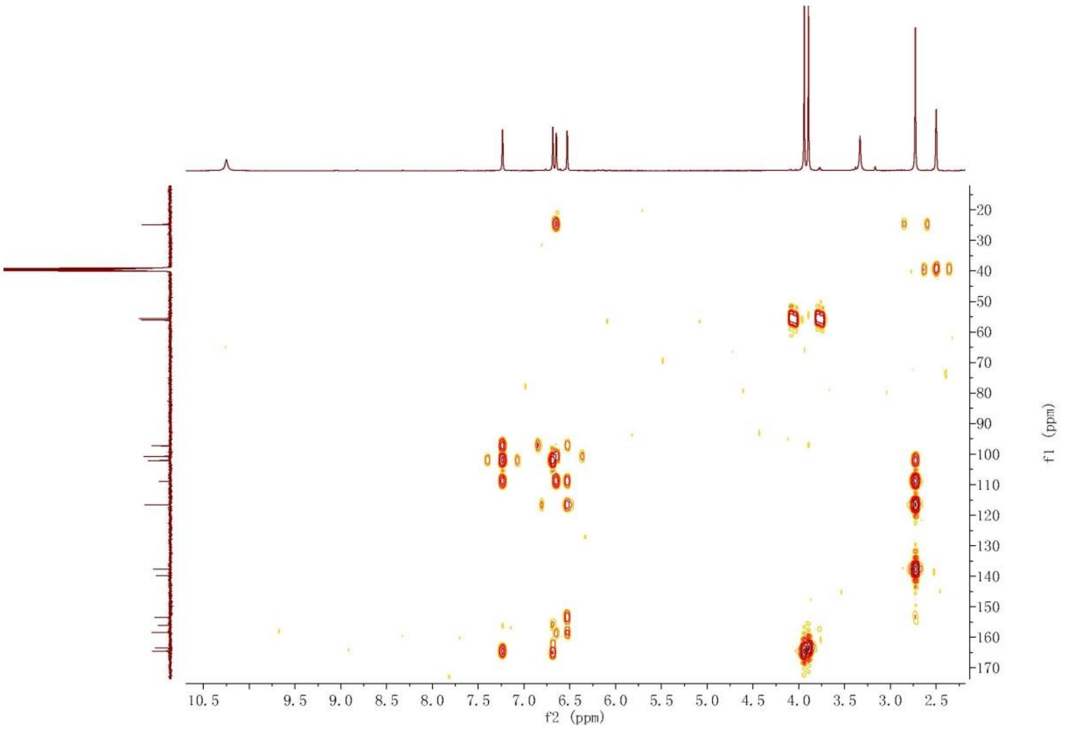

^{1}H- and ^{13}C-NMR Data of Compound 4

Colorless oil; ^{1}H NMR data (at 500 MHz in DMSO-d_6): δ_H 4.18 (1H, ddd, J = 9.0, 4.1, 0.7 Hz, H-2), 2.33 (1H, m, H-3a), 1.98 (1H, m, H-3b), 2.14 (2H, m, H-4), 7.98 (1H, br s, H-6), 3.67 (3H, s, H-7); ^{13}C NMR data (at 125 MHz in DMSO-d_6): δ_C 173.3 (C-1, C), 54.6 (C-2, CH), 24.4 (C-3, CH2), 28.8 (C-4, CH2), 176.9 (C-5, C), 52.0 (C-7, CH3).

^{1}H- and ^{13}C-NMR Data of Compound 5

Yellow oil; $[\alpha]_D^{27}$ +90 (c 0.10, MeOH); ^{1}H NMR data (at 500 MHz in DMSO-d_6): δ_H 3.48 (2H, m, H-3), 1.88 (2H, m, H-4), 2.21 (1H, m, H-5a), 1.94 (1H, m, H-5b), 4.44 (1H, dd, *J* = 9.2, 7.1 Hz, H-6), 6.94 (1H, s, H-10), 2.73 (3H, s, H-11), 7.43-7.31 (5H, m, H-2′, 3′, 4′, 5′, 6′); ^{13}C NMR data (at 125 MHz in DMSO-d_6): δ_C 167.7 (C-1, C), 44.8 (C-3, CH2), 22.2 (C-4, CH2), 27.4 (C-5, CH2), 58.0 (C-6, CH), 160.0 (C-7, C), 134.2 (C-9, C), 118.6 (C-10, CH), 34.1 (C-11, CH3), 133.6 (C-1′, C), 129.4 (C-2′, CH), 128.3 (C-3′, CH), 128.1 (C-4′, CH), 128.3 (C-5′, CH), 129.4 (C-6′, CH).

^{1}H- and ^{13}C-NMR Data of Compound 6

Yellow oil; ^{1}H NMR data (at 500 MHz in DMSO-d_6): δ_H 3.52 (1H, dd, *J* = 12.5, 4.7 Hz, H-3a), 3.16 (1H, m, H-3b), 4.19 (1H, m, H-4), 1.94 (1H, dd, *J* = 12.9, 6.3 Hz, H-5a), 1.52 (1H, m, H-5b), 4.29 (1H, dd, *J* = 11.0, 6.2 Hz, H-6), 7.98 (1H, br s, H-8), 4.40 (1H, t, *J* = 4.9 Hz, H-9), 3.08 (1H, dd, *J* = 14.2, 5.3 Hz, H-10a), 3.01 (1H, dd, *J* = 14.2, 5.3 Hz, H-10b), 5.09 (1H, d, *J* = 2.4 Hz, 4-OH), 7.21-7.17 (5H, m, H-2′, 3′, 4′, 5′, 6′); ^{13}C NMR data (at 125 MHz in DMSO-d_6): δ_C 170.7 (C-1, C), 54.0 (C-3, CH2), 66.6

(C-4, CH), 37.4 (C-5, CH2), 56.8 (C-6, CH), 165.4 (C-7, C), 55.4 (C-9, CH), 35.3 (C-10, CH2), 137.4 (C-1′, C), 129.7 (C-2′, CH), 128.0 (C-3′, CH), 126.3 (C-4′, CH), 128.0 (C-5′, CH), 129.7 (C-6′, CH).

^{1}H- and ^{13}C-NMR Data of Compound 7

Yellow oil; ^{1}H NMR data (at 500 MHz in DMSO-d_6): δ_H 3.40 (1H, m, H-3a), 3.33 (1H, m, H-3b), 1.80 (2H, m, H-4), 2.12 (1H, m, H-5a), 1.84 (1H, m, H-5b), 4.13 (1H, m, H-6), 7.96 (H, br s, H-8), 3.91 (1H, s, H-9), 2.34 (1H, m, H-10), 1.01 (3H, d, *J* =7.2 Hz, H-11), 0.84 (3H, d, *J* = 6.8 Hz, H-12); ^{13}C NMR data (at 125 MHz in DMSO-d_6): δ_C 170.2 (C-1, C), 44.6 (C-3, CH2), 22.0 (C-4, CH2), 27.8 (C-5, CH2), 58.2 (C-6, CH), 165.2 (C-7, C), 59.4 (C-9, CH), 27.7 (C-10, CH), 18.3 (C-11, CH3), 16.4 (C-12, CH3).

^{1}H- and ^{13}C-NMR Data of Compound 8

Yellow amorphous powder; ^{1}H NMR data (at 500 MHz in DMSO-d_6): δ_H 6.72 (1H, s, H-3), 6.38 (1H, s, H-7), 3.42 (3H, s, H-8), 2.05 (3H, s, H-9); ^{13}C NMR data (at 125 MHz in DMSO-d_6): δ_C 168.7 (C-1, C), 101.0 (C-3, CH), 121.7 (C-3a, C), 151.5 (C-4, C), 118.7 (C-5, C), 158.8 (C-6, C), 102.1 (C-7, CH), 124.8 (C-7a, C), 55.3 (C-8, CH_3), 9.1 (C-9, CH_3).

^{1}H- and ^{13}C-NMR Data of Compound 9

Yellow oil; ^{1}H NMR data (at 500 MHz in DMSO-d_6): δ_H 6.50 (1H, d, *J* = 2.3 Hz, H-4), 6.67 (1H, d, *J* = 2.3 Hz, H-6), 2.26 (1H, dd, *J* = 14.1, 3.3 Hz, H-3′a), 1.95 (1H, dd, *J* = 14.1, 7.4 Hz, H-3′b), 3.69 (1H, m, H-4′), 3.95 (1H, s, H-5′), 5.15 (1H, d, *J* = 3.6 Hz, 4′-OH), 5.31 (1H, d, *J* = 6.1 Hz, 5′-OH), 6.30 (1H, d, *J* = 3.2 Hz, H-6′), 1.47 (3H, s, 2′-CH_3), 3.86 (3H, s, 5-OCH_3), 11.30 (1H, br s, 3-OH); ^{13}C NMR data (at 125 MHz in DMSO-d_6): δ_C 131.0 (C-1, C), 99.8 (C-2, C), 165.8 (C-3, C), 100.9 (C-4, CH), 163.0 (C-5, C), 55.8 (5-OCH_3), 102.0 (C-6, CH), 168.2 (C-7, C), 26.2 (2′-CH_3), 139.2 (C-1′, C), 81.1 (C-2′, C), 38.6 (C-3′, CH_2), 68.7 (C-4′, CH), 69.5 (C-5′, CH), 131.2 (C-6′, CH).

^{1}H- and ^{13}C-NMR Data of Compound 10

Yellow oil; ^{1}H NMR data (at 500 MHz in DMSO-d_6): δ_H 6.50 (1H, d, *J* = 2.3 Hz, H-4), 6.74 (1H, d, *J* = 2.3 Hz, H-6), 2.16 (1H, dd, *J* = 11.9, 3.6 Hz, H-3′a), 2.03 (1H, t, *J* = 11.9 Hz, H-3′b), 3.61 (1H, m, H-4′), 4.09 (1H, m, H-5′), 5.22 (1H, d, *J* = 5.0 Hz, 4′-OH), 5.38 (1H, d, *J* = 6.1 Hz, 5′-OH), 6.25 (1H, d, *J* = 2.4 Hz, H-6′), 1.49 (3H, s, 2′-CH_3), 3.86 (3H, s, 5-OCH_3), 11.34 (1H, br s, 3-OH); ^{13}C NMR data (at 125 MHz in DMSO-d_6): δ_C 131.8 (C-1, C), 100.0 (C-2, C), 165.8 (C-3, C), 101.2 (C-4, CH), 163.2 (C-5, C), 55.8 (5-OCH_3), 102.3 (C-6, CH), 167.8 (C-7, C), 27.4 (2′-CH_3), 137.6 (C-1′, C), 82.2 (C-2′, C), 43.4 (C-3′, CH_2), 70.2 (C-4′, CH), 72.5 (C-5′, CH), 131.0 (C-6′, CH).
